# In vivo gene editing of *CAMKIID*: out with the bad and in with the good

**DOI:** 10.1172/JCI176672

**Published:** 2024-01-02

**Authors:** John E. Smith, Henk Granzier

**Affiliations:** Department of Cellular and Molecular Medicine, The University of Arizona, Tucson, Arizona, USA.

## Abstract

The ability to change an organism’s DNA through gene editing is of great importance for the prevention and treatment of genetic and acquired diseases. Rapid progress has been made during the last decade due to the discovery and refinement of CRISPR/Cas9 as an accurate, fast, and reliable genome editing technique. In this issue of the *JCI*, Lebek et al. present the culmination from a line of work in the Olson laboratory focused on in vivo gene editing of *CAMK2D*. The paper presents a combined state-of-the-art gene therapy approach that demonstrates how gene therapy can yield cardioprotection in a mouse model and takes notable steps toward potential applicability in patients.

## CAMK2D

The *CAMK2D* gene encodes calcium/calmodulin dependent protein kinase II delta (CaMKIIδ), one of the major CaMKII isoforms and the predominant isoform in the heart. CaMKIIδ contributes to the cardiac myocyte’s excitation-contraction coupling by governing the activity of proteins involved in calcium flux, such as ryanodine receptors, phospholamban, and L-type Ca^2+^ channels. It is also involved with the phosphorylation of sarcomeric proteins, including titin (1, 2). The function of CaMKIIδ can be modulated in response to alterations in intracellular calcium levels and through various posttranslational modifications. Some effects attributed to CaMKIIδ may be seen as adaptive, while sustained CaMKIIδ activity can have detrimental consequences for the structure and function of the heart (2). Studies conducted in the Anderson laboratory utilizing knockin mouse models have shown that CaMKIIδ features a regulatory domain with two methionine residues (M281 and M282) susceptible to oxidation (3). These methionines, when oxidized, have been associated with various pathological conditions, including myocardial infarction and ischemia/reperfusion (IR) injury.

## Gene targeting *CAMK2D* and IR injury

In this issue of the *JCI*, Lebek and colleagues (4) aimed to utilize the CRISPR Cas9 adenine base editing technique to edit *CAMK2D* in vivo, with the objective of substituting the vulnerable methionines, M281 and M282, with oxidation-resistant valines. Utilizing a mouse surgery model of IR injury, the authors injected the components for editing CaMKIIδ directly into the heart. Three weeks after IR, mice with edited *CAMK2D* exhibited enhanced left ventricular function, as revealed by the increased fractional shortening and much-improved chamber dimensions in diastole. That these functional parameters are meaningful at the level of the individual was shown by the improved exercise performance that the authors studied 4 weeks after IR. Mice that had only a single methionine converted to valine (M281) were protected from injury but to a lesser degree compared with mice in which both methionines were targeted (M281 and M282). When both sites were targeted, the treatment prevented the development of fibrosis during the period after IR, and changes in heart weight and body weight were absent. Thus, a superior degree of protection was achieved from the damaging effect of IR injury, following gene editing of both M281 and M282.

Lebek and colleagues (4) made multiple technical breakthroughs to achieve this remarkable outcome. First, they developed a mouse model in which the regulatory domain of *CAMK2D* was humanized. This model makes it possible to optimize gene editing strategies in mice for ultimate use in patients with heart disease. Additionally, the study developed targeting strategies for modifying one methionine versus both methionines. To minimize off-target edits, an adenine base editor was used; and to circumvent the AAV-packaging limit, a dual vector split-intein trans-splicing system was employed. Notably, as elaborated by Lebek et al. (4), the authors also took advantage of editing strategies that introduced a mutation in the base editor that further minimized off-target editing (5). Finally, the authors optimized a recently developed MyoAAV 2A delivery system (6) that demonstrated superior cardiac transduction efficiency requiring just a single editing dose and triggering substantially fewer adverse side effects compared with conventional AAV delivery methods. All of these steps are major advances that bring us closer to offering cardio-protection against oxidative damage in patients.

There are critical next steps to consider. Injecting the gene editing components directly into the heart, as was done in the Lebek et al. study (4), would pose challenges in clinical settings, and alternative delivery methods need to be investigated. Additionally, in the real world, myocardial infarction is typically not immediately detected, and treatments cannot be promptly administered. Therefore, comprehensive follow-up studies are essential, examining variations in the timing of treatment relative to the ischemic event, to establish the gene editing time window within which a positive outcome can be achieved. Moreover, although the mice displayed minimal noticeable side effects 5 weeks after cardiac intervention, the exploration of potential long-term side effects, both in the heart and other tissues, remains a critical area for further research. Considering that the targeted residues (M281 and M282) are well conserved, they might perform important functions under some type of physiological condition or during a certain developmental stage (7). Thus, long-term follow-up studies are warranted.

## Implications and conclusion

Building on a wealth of insights in the role of CaMKIIδ in IR injury accumulated by many investigators and making a host of critically important technological developments for successful in vivo gene editing, Lebek et al. (4) convincingly show that cardioprotective gene therapy can be achieved in vivo, using a mouse model of IR injury. Although challenges remain before similar approaches can be applied to patients with heart disease, the present work lends confidence that this might be possible in the near future. Considering that CaMKIIδ has been linked to multiple disorders and that the approaches pioneered by Lebek et al. (4) are applicable to other clinically relevant genes, the present study paves the way for important follow-up work addressing a wide range of diseases.

## Version 1 01/02/2024

Electronic publication
